# Complete Chloroplast Genome of *Argania spinosa*: Structural Organization and Phylogenetic Relationships in Sapotaceae

**DOI:** 10.3390/plants9101354

**Published:** 2020-10-13

**Authors:** Slimane Khayi, Fatima Gaboun, Stacy Pirro, Tatiana Tatusova, Abdelhamid El Mousadik, Hassan Ghazal, Rachid Mentag

**Affiliations:** 1 CRRA-Rabat, National Institute for Agricultural Research (INRA), Rabat 10101, Morocco; fatima.gaboun@inra.ma; 2 Iridian Genomes, Inc., Bethesda, MD 20817, USA; info@iridiangenomes.org; 3 National Center for Biotechnology Information, National Institutes of Health, Bethesda, MD 20817, USA; tatiana@ncbi.nlm.nih.gov; 4 Laboratory of Biotechnology and Valorization of Natural Resources (LBVRN), Faculty of Sciences, University Ibn Zohr, Agadir 80000, Morocco; a.elmousadik@uiz.ac.ma; 5 National Center for Scientific and Technological Research (CNRST), Rabat 10102, Morocco; hassan.ghazal@fulbrightmail.org

**Keywords:** chloroplast genome, sapotaceae, *Argania spinosa*, phylogenomic analysis, molecular marker

## Abstract

*Argania spinosa* (Sapotaceae), an important endemic Moroccan oil tree, is a primary source of argan oil, which has numerous dietary and medicinal proprieties. The plant species occupies the mid-western part of Morocco and provides great environmental and socioeconomic benefits. The complete chloroplast (cp) genome of *A. spinosa* was sequenced, assembled, and analyzed in comparison with those of two Sapotaceae members. The *A. spinosa* cp genome is 158,848 bp long, with an average GC content of 36.8%. The cp genome exhibits a typical quadripartite and circular structure consisting of a pair of inverted regions (IR) of 25,945 bp in length separating small single-copy (SSC) and large single-copy (LSC) regions of 18,591 and 88,367 bp, respectively. The annotation of *A. spinosa* cp genome predicted 130 genes, including 85 protein-coding genes (CDS), 8 ribosomal RNA (rRNA) genes, and 37 transfer RNA (tRNA) genes. A total of 44 long repeats and 88 simple sequence repeats (SSR) divided into mononucleotides (76), dinucleotides (7), trinucleotides (3), tetranucleotides (1), and hexanucleotides (1) were identified in the *A. spinosa* cp genome. Phylogenetic analyses using the maximum likelihood (ML) method were performed based on 69 protein-coding genes from 11 species of *Ericales*. The results confirmed the close position of *A. spinosa* to the *Sideroxylon* genus, supporting the revisiting of its taxonomic status. The complete chloroplast genome sequence will be valuable for further studies on the conservation and breeding of this medicinally and culinary important species and also contribute to clarifying the phylogenetic position of the species within Sapotaceae.

## 1. Introduction

The argan tree (*Argania spinosa L. Skeels*) is an endemic plant species of the middle west of Morocco and the unique member of the tropical Sapotaceae family in this Mediterranean country [1]. In 1999, UNESCO classified the argan tree as a world heritage. Extracted from seeds, argan oil is the worldwide precious product of the argan tree, used as edible or cosmetic oil [2]. Thus, this forest fruit and forage species is the backbone of a traditional arganian system that has hitherto served the needs of a dense population in an arid zone. Unfortunately, due to the pressure of several factors such as human overexploitation and climate change, the Argan forest was drastically deteriorated during the 18th century, and about 44% of the forest was lost between 1970 and 2007 [3,4]. Therefore, the management and conservation of the remaining genetic resources of this species are urgent priorities.

The Sapotaceae family is composed of about 50 genera and 1100 species which are distributed in the tropical regions with some exception, especially, *A. spinosa*, which occupies the mid-western part of Morocco [5]. Previous biogeography and phylogenetic analyses of Sapotaceae species based on different elements, such as several chloroplast genes [6,7,8], the chloroplast *ndhF* gene combined with morphological data [9], and nuclear ITS (Internal Transcribed Spacer) combined to the chloroplast *trnH–psbA* regions, have been reported [10]. These phylogenetic studies, inferred using few genetic makers, have strongly recommended that the satellite genus of *Argania* must be included into the genus of *Sideroxylon*, hence revisiting the phylogenetic status of *A. spinosa*. Thus, more genetic markers are needed to clarify this phylogenetic revision which still remains debatable.

During the last two decades, the advent of next-generation sequencing (NGS) technologies has accelerated the pace of deciphering the chloroplast (cp) genomes of many plant species; presently, 3949 land plant cp genomes have been deposited in GenBank Organelle Genome Resources (accessed on 25 June 2020). This photosynthetic organelle provides essential energy for plants and algae and represents a valuable resource for exploring intra- and inter-specific evolutionary histories of land plants [11,12,13,14,15]. In addition, due to their several characteristics such as small length, simple structure, maternal inheritance characters, conserved sequences, and very low level of recombination [16], the chloroplast genomes are commonly used for studies of plant evolution, phylogeny, and traceability [17,18]. To date, two full-length chloroplast sequences have been assembled within Sapotaceae, i.e., those of *Sideroxylon wightianum* (MG719834) and *Pouteria campechiana* (MH018545.1). A comprehensive phylogenetic analysis based on whole cp genomes should help to more accurately elucidate the phylogenetic status of *A. spinosa*. A first draft nuclear genome of *A. spinosa* has recently been published [19] and deposited in GenBank (QLOD00000000.1), but little is known about its cp genome structure, except from the work of El Mousadik and Petit [20] who studied the phylogeography of argan tree using universal chloroplast primers targeting specific chloroplast genes. 

In the present study, we report the complete cp genome sequence of *A. spinosa*, assembled from Illumina short reads for the first time and compare the genomes of three Sapotaceae species to understand the variations among their cp genomes. The objectives of this study were: (i) assemble and depict the whole cp genome structure of *A. spinosa*, (ii) perform extensive comparative genomics with other Sapotaceae cp genomes, (iii) report the simple sequence repeats in cp genomes to provide tools for future genetic diversity and breeding studies, and lastly iv) assess the taxonomic positions of *A. spinosa* based on its complete cp genome.

## 2. Results and Discussion

### 2.1. Genome Assembly

Illumina sequencing of two libraries generated a total of 957,451,810 (SRR6062046, SRR6062045) raw reads. After a quality processing step, 936,053,040 reads were mapped against the cp reference genome to collect the *A. spinosa* cp-like reads. Paired-end reads were then extracted from the mapping, yielding 7,9724,230 sequences representing 8.5% of the total whole genome shotgun data. De novo assembly with CLC genomics (v11.0, CLCbio, Arhus, Denmark) generated 23 contigs with the maximum length of 39 kbp. The alignment of the contigs against the cp reference genome resulted in seven contigs, totaling 158,281 bp. The aligned contigs were assembled into one chromosome by the Genome Finishing Module (GFM) from CLC genomics using *S. wightianum* cp genome as a reference. 

**Figure 1:**
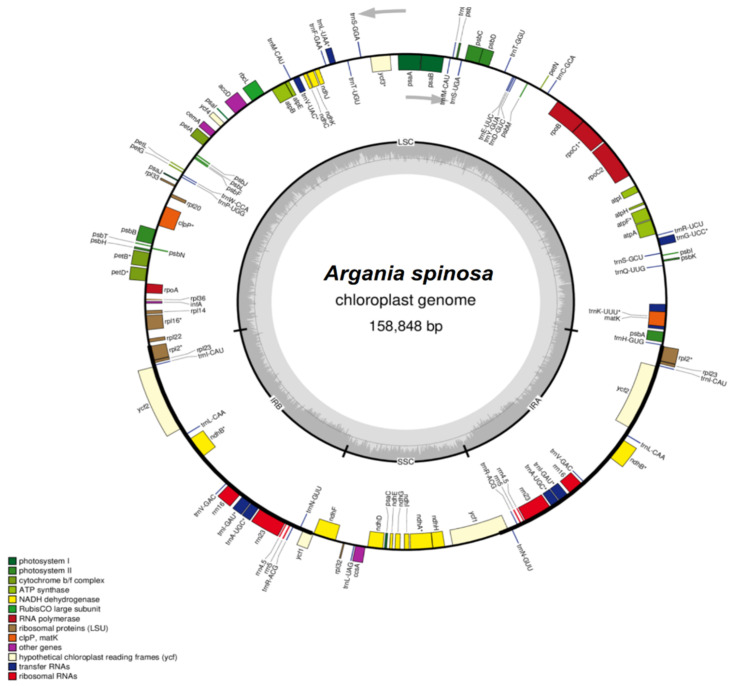
Gene map of *Argania spinosa* chloroplast genome. The thick lines colored in black in the inner circle indicate the extent of the inverted repeat regions (IRa and IRb; 25,946 bp), which separate the genome into small (SSC; 18,593 bp) and large (LSC; 88,367 bp) single-copy regions. Genes drawn inside the circle are transcribed clockwise, and those on the outer side are transcribed counter-clockwise. Genes belonging to different functional groups are color-coded. The dark grey in the inner circle corresponds to the GC content, and the light grey corresponds to the AT content.

The *A. spinosa* genome consists of a circular molecule measuring 158,848 bp in length, with 36.9% GC content, which is consistent with other sequenced cp genomes of the Sapotaceae family, whose plastome GC content was 36.8% and 38.9% for *P. campechiana* and *S. wightianum*, respectively. The whole genome alignment to the cp reference and the dot plot of the genome sequence confirmed the quadripartite structure found in most chloroplast genomes of plants [12,14,15,21,22] (Figure 1). The genome has an inverted repeat (IR) region 25,945 bp in length, a large single-copy (LSC) region of 88,367 bp, and a small single-copy (SSC) region of 18,591 bp. The GC content was 42% in the IR region and 34% and 30% in the LSC and SSC regions, respectively, at relatively the same level as in *S. wightianum* (IR 42%, LSC 34%, SSC 30%) and *P. campechiana* (IR: 42%, LSC: 34%, SCC: 30%). The high GC content registered in the IR regions is mainly due to the high GC contents of the four ribosomal RNA (rRNA) genes *rrn4.5*, *rrn5*, *rrn16*, *rrn23* that are located in the IR regions and display, respectively, 50%, 52%, 56%, and 54% of GC content.

The validation of the assembly was performed by PCR and Sanger sequencing using four couples of primers designed on the boundaries of the IR and single-copy regions (Table S1). PCRs were performed using the DNA extracts of four different individuals of *A. spinosa* (V1, V2, V3, and V4). The A and D couple of primers targeted the IRa/LSC and IRa/SSC junctions and amplified, respectively, 700 and 258 bp fragments, as shown in Figure S1. The B and C primers were designed to verify the IRb/SSC and IRb/LSC junctions, respectively, amplifying fragments of 699 and 361 bp in length (Figure S1).

### 2.2. Features of the A. spinosa Chloroplast Genome

The annotation process predicted a total of 130 functional genes representing 85 protein-coding genes, 37 tRNAs and 8 rRNAs. The coding domain sequences (CDSs) account for 80,967 bp in length, which represents 50.97% of *A. spinosa* cp genome. The gene proportion for tRNA is 1.75%, and that for *rRNA* is 5.69%. The proportion of non-coding regions, which contain intergenic spacers and introns, represents 49.02% of the cp genome. The protein-coding sequences include 6 duplicated genes (*rpl2*, *nhB*, *rpl23*, *rps7*, *ycf2*, and *rps12*), 1 pseudogene (*Ψycf1)*, 4 rRNAs in two copies, and 37 tRNAs with 7 duplicated genes (*trnA-UGC*, *trnI-CAU*, *trnI-GAU*, *trnL-CAA*, *trnN-GUU*, *trnV-GAC*, and *trnR-ACG*). The IR regions contains six CDS (*rpl2, rpl23*, *ycf2*, *ndhB*, *rps7*, *rps12*), four rRNAs and seven tRNAs. The SSC region contains 12 CDS and 1 tRNA, while the LSC region harbors 60 CDS and 22 tRNAs (Table 1, Figure 1). 

The *A. spinosa* cp genome was found to contain introns in some annotated genes, like other cp genomes of angiosperms [23,24]. A total of nine protein-coding genes (*rpl2, ndhB, rps12, ndhA, rps16*, *petD*, *petB*, *rpoC1*, *atpF*) and six tRNA genes contained a single intron, while three genes (*ycf3, clpP¡*, and *rps12*) contained two introns. The *rps*12 gene was predicted to be trans-spliced, with the 5′ end located in the LSC region and the duplicated 3′ end in the IR region. The *trnK-UUU* gene has the longest intron (2535 bp) that contains coding sequences of the *matK* gene, whereas the intron of *trnL-UAA* is the smallest (509 bp). The complete cp genome was deposited in Organelle Genome Resources (GenBank accession MK533159).

**Figure 2:**
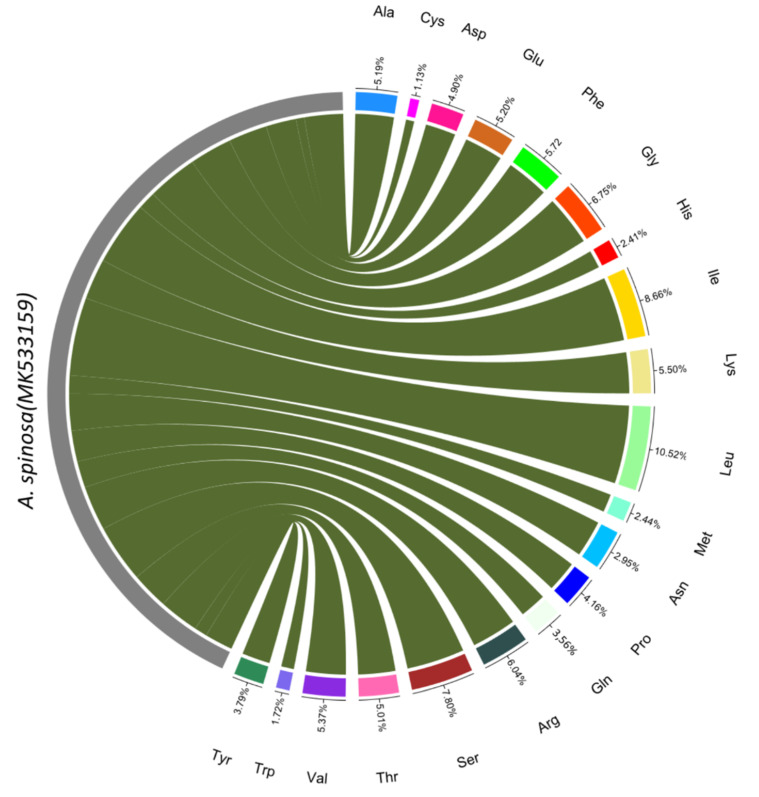
Amino acid frequencies of the *A. spinosa* chloroplast protein-coding sequences. The frequencies of amino acids were calculated for all 85 protein-coding genes from the start to the stop codons.

The sequences of tRNA and protein-coding genes were analyzed, and the codon-usage frequency was calculated for *A. spinosa* (Table S2). In total, 26,799 codons were identified for 83 protein-coding sequences in *A. spinosa* cp genome. The use of the codons ATG and TGG, which encode, respectively, Methionine and Tryptophan, exhibited no bias (Relative Synonymous Codon Usage, RSCU = 1). The maximum AUU (1078) and the minimum CGC (104) codons used coded for isoleucine and arginine, respectively, and the ending bases A and U were preferred in the synonymous codon (RSCU > 1). However, for the non-preferred synonymous codons, the ending bases were G or C. The same phenomenon was described in previous studies of cp genomes [14,25]. The frequencies of amino acids for the protein-coding sequence were calculated for *A. spinosa* (Figure 2). Leucine represents the most frequent amino acid in the *A. spinosa* cp genome, with 2586 codons (10.3%). Cysteine is the least frequent amino acid, with only 450 codons (1.3%). Similar ratios for amino acids were reported in previous studies [26,27]. 

### 2.3. Comparative Analysis of cp Genome Structures

**Figure 3:**
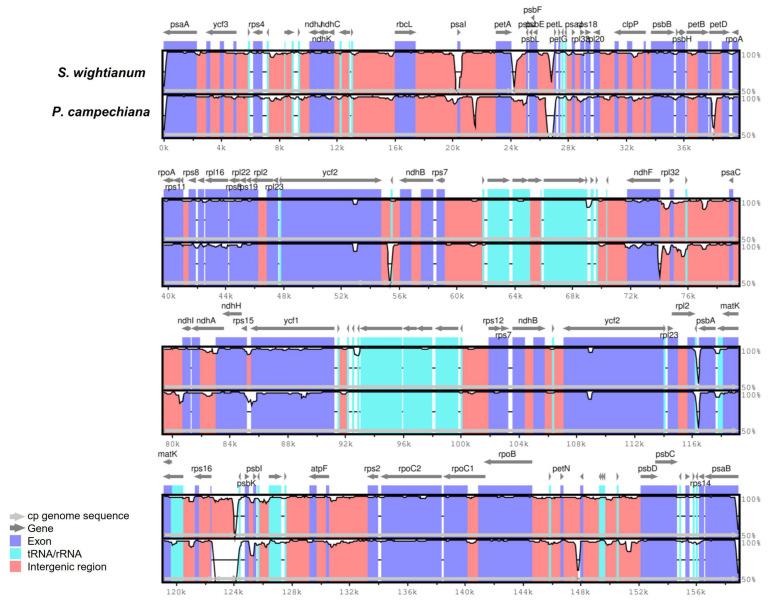
mVISTA percent identity plot comparing the three Sapotaceae plastid genomes with *A. spinosa* as a reference. The top line shows genes in order (transcriptional direction indicated by arrows). The y-axis represents the percent identity within 50–100%. The x-axis represents the coordinate in the chloroplast genome. Genome regions are color-coded as protein-coding (exon), tRNAs or rRNAs, and conserved noncoding sequences (intergenic region). The white block represents regions with sequence variation between the two species.

To understand the structural characteristics of the cp genomes of Sapotaceae sequenced to date, overall sequence alignment of the three cp genome sequences was conducted using the annotation of *A. spinosa* as a reference (Figure 3). The aligned sequences appeared to be relatively conserved, with a slight level of sequence divergence in some regions. The gene-coding regions are more highly conserved than those of their non-coding counterparts and intergenic regions, which is consistent with the pattern reported for several angiosperm cp genomes [22]. The most divergent sequences were found within the intergenic spacers and introns of these three sequences, including *rbcL–psaI*, *psaI–petA*, *psbE–petL*, *ycf2–trnL-CAA*, *rpl32–ndhF*, *ycf1–rpS15*, *psbA–trnH-GUG*, *trnQ-UUG–rpS16*, *psbI–psbK, psbM–petN*. In the coding regions, slight variations in sequences were observed in *petD*, *ndhA*, and *ycf2*. Most of these hotspot regions are located in the LSC regions, and only few regions are located in the SSC or IR regions, as shown in Figure 3.

**Figure 4:**
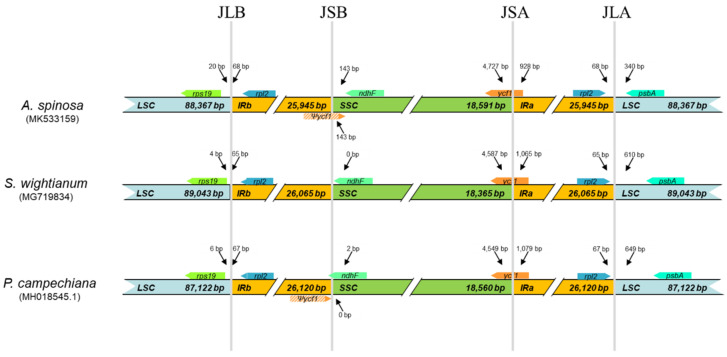
A comparison of the distance between adjacent genes and junctions in the SSC, LSC, and two IR regions for the chloroplast genomes of *A. spinosa*, *Sideroxylon wightianum*, and *Pouteria campechiana*. The figure shows relative changes at or near the IR/SC borders, with no scale to sequence length. The colored boxes indicate genes.

Inverted repeat regions are variable in land plants’ cp genomes, ranging from a couple of hundred [28] to several thousand [13] base pairs in size. It is reported that large IR play a key role in genome stability of chloroplasts [29]. A detailed comparison of the four junctions LSC–IRa (JLA), LSC–IRb (JLB), SSC–IRa (JSA), and SSC–IRb (JSB) of three *Sapotaceae* cp genomes (*A. spinosa*, *S*. *wightianum*, and *P. campechiana*) is presented in Figure 4. Although the IR regions of the three cp genomes were highly conserved with slight variations. We found five genes, *rps19*, *ycf1*, *ndhF*, *rpl2*, and *psbA*, among the three species to be implicated in the four junctions. The *rps19* gene is located in the LSC region at 20, 4, and 6 bp from the JLB border, and the gene *psbA* is located in the same region at 340, 610, and 649 bp from JLA in these three *Sapotaceae* cp genomes, respectively. The *rpl2* gene is located in the IR region at 68, 65, and 67 bp from the JLA/JLB junctions, respectively, in the three cp genomes. The gene *ndhF*, located in the SSC, was found to end exactly at the JSB junction in *S. wightianum* genome and to cross the junction by 2 bp in *P. campechiana* genome. However, the distance between this junction and *ndhF* was of 143 bp for *A. spinosa*. The pseudogene *ycf1*, that was not predicted in *S. wightianum*, was found to cross the JSB junction by 143 bp in *A. spinosa* genome and to end at the limit of this junction in *P. campechiana* genome. The JSA junction was crossed by the gene *ycf1* in the three cp genomes, and the fragment located in the IRa region ranged from 928 to 1079 bp. These results showed the slight contraction of the IR region in *A. spinosa* cp genome.

### 2.4. Nucleotide Diversity and Divergence of Coding Gene Sequences

**Figure 5:**
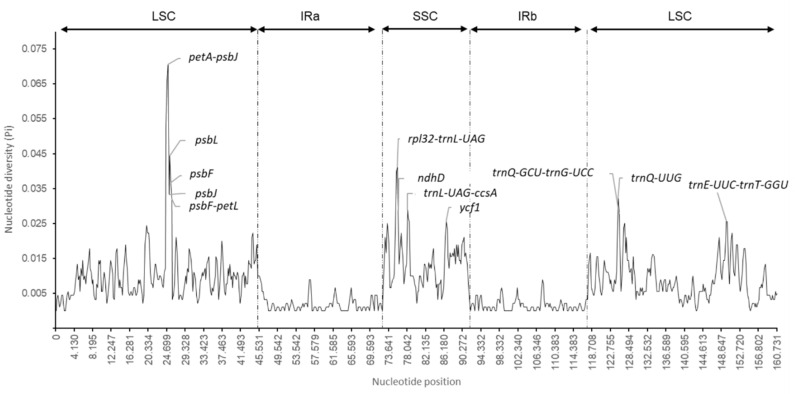
Nucleotide diversity (Pi) values for the whole chloroplast genomes of *A. spinosa*, *S. wightianum*, and *P. campechiana* species using a window length of 600 bp and a step size of 200 bp.

Nucleotide diversity indices (Pi), determined using DnaSP and calculated for the three species using a window of 600 bp, showed Pi values ranged from 0 to 0.070, with an average of 0.007, indicating that the divergence between the genomes is small. As described in previous studies [13,25], the IR regions had a much lower nucleotide variability (Pi = 0.001785) than the SSC (Pi = 0.01362) and LSC (Pi = 0.010471) regions (Table S3, Figure 5). Based on this analysis, 14 midpoints of sliding windows showed high levels of nucleotide diversity, with Pi values > 0.025, corresponding to *petA–psbJ*, *psbJ*, *psbL*, *psbF*, *psbF–petL*, *rpl32–trnL-UAG*, *trnQ-UUG*, *ndhF*, *trnL-UAG–ccsA*, *ndhD*, *ycf1*, *trnQ-GCU–trnG-UCC*, *trnG-UCC–trnR-UCU*, *trnE-UUC–trnT-GGU*. Most of these highly variable regions are found in intergenic spacers in LSC and SSC regions. Therefore, these highly variables regions in cp genomes can be useful for phylogenetic reconstruction of the large family of Sapotaceace.

**Figure 6:**
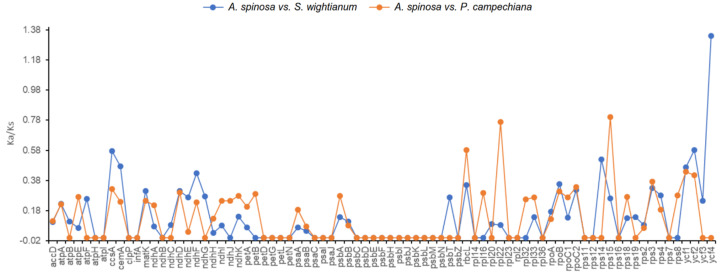
The Ka/Ks values of 79 protein-coding genes of the three Sapotaceae cp genomes. The calculation was performed for paired sequences of the species *A. spinosa* vs. *S. wightinaum* and *A. spinosa* vs. *P. campechiana*.

To elucidate the selective pressure on the 79 genes in common among the 3 cp genomes, the rates of synonymous (Ks) and nonsynonymous (Ka) substitutions and the Ka/Ks values were calculated (Table S4, Figure 6). The Ka/Ks values may indicate whether selective pressure occurred for plastid genes. Thus, Ka/Ks < 1 suggests that a cp DNA gene was under purifying selection, whereas Ka/Ks ≥ 1 indicates that the gene was affected by positive selection or neutral selection [30].

The lowest Ka/Ks ratio was observed for genes encoding NADH (Nicotinamide Adenine Dinucleotide Hydrogen) dehydrogenase, i.e., *ndhE* (Ka/Ks = 0.03) and *ndhD* (Ka/Ks = 0.3), for the paired species *A. spinosa/S. wightianum* and *A. spinosa/P. campechainana*, respectively. The highest Ka/Ks ratios were calculated for *rps15* (Ka/Ks = 0.8) and *ycf4* (Ka/Ks = 1.3) genes for *A. spinosa/S. wightianum* and *A. spinosa/P. campechainana*, respectively.

The Ka/Ks ratio was found to be 0 for 47 genes, the majority of which is located in the LSC region, for the two paired species *A. spinosa/S. wightianum* and *A. spinosa/P. campechainana* (Table S4). For these genes, the *K*a/*K*s values could not be calculated because *K*a or *K*s was extremely low or equal to 0 [31,32]. The remaining 38 genes, mostly located in the LSC region, showed Ka/Ks ratios below 1.00, indicating a purifying selection. The Ka/Ks ratio was found to indicate positive selection for only one gene, *ycf4* (Ka/Ks = 1.3), for the paired sequence species *A. spinosa/S. wightianum*. Similar results were reported for other cp genomes [33,34,35].

### 2.5. Long Repeat and Simple Sequence Repeats (SSR) Analysis

The analysis of long repeats within the *A. spinosa* cp genome showed a total of 44 repeats represented by 18 forward repeats, 23 palindromic repeats, 2 reverse repeats, and 1 complement repeat. Out of 44 repeats within *A. spinosa* cp genome, 30 repeats (69%) were 30–39 bp long, 8 repeats (18%) were >50 bp long, and 6 repeats (13%) were 40–49 bp long (Table S5). 

**Figure 7:**
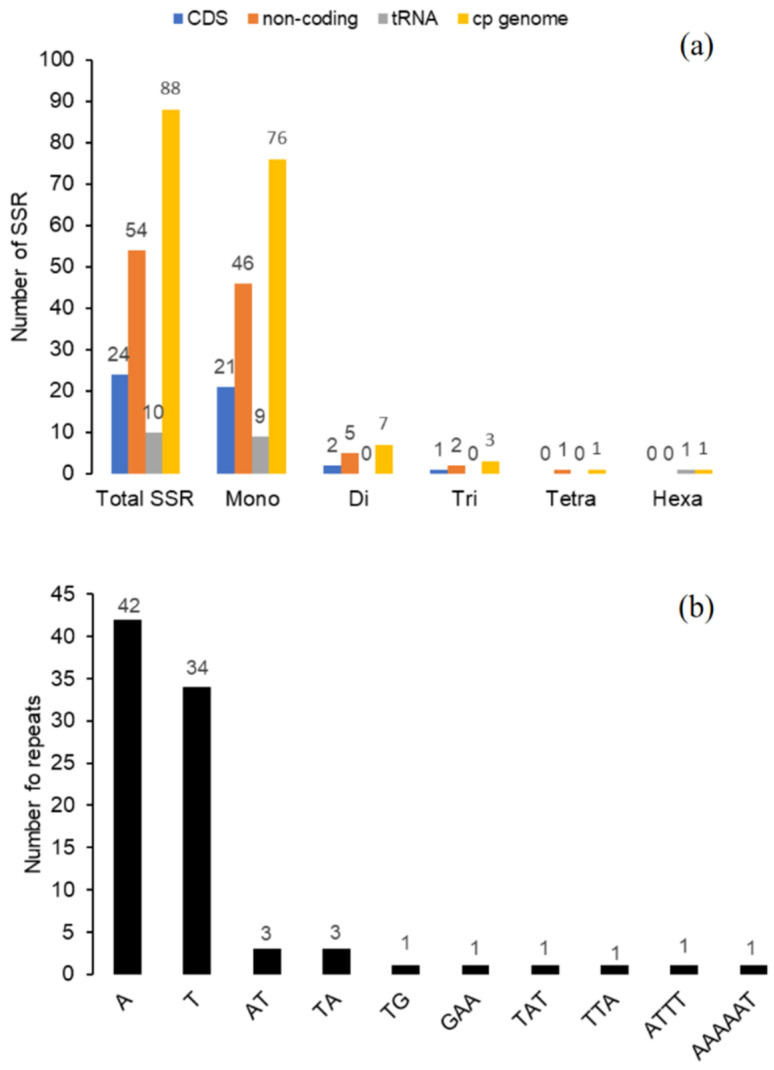
Analysis of simple sequence repeats (SSRs) in *A. spinosa* cp genome. (**a**) Number of SSR types in the cp complete genome, coding, and non-coding regions; (**b**) Number and type of SSR motifs identified in the cp genome. CDS, coding domain sequences.

SSRs, also known as microsatellites, are short tandemly repeated sequences of typically 1 to 6 nucleotides repeat units [36]. They are widely distributed in cp genomes, are important for plants population studies because of their high level of polymorphism compared to neutral DNA regions, and are uniparentally inherited. SSRs have been widely used as molecular markers for variety/species identification, molecular breeding, and genetic diversity assessment [37,38]. In this study, SSRs distribution within the *A. spinosa* cp genome was determined. Using the MISA software, the analysis highlighted a total of 88 SSRs composed of 76 mononucleotides SSRs, 7 dinucleotides, and 3, 1, 1 tri-, tetra-, and hexanucleotide, respectively (Figure 7a). The highest number of mononucleotide SSRs were A (47.7%) and T (38.6%) motifs, and most dinucleotide SSRs were found to be AT/TA (6.8%) and TG (1,13) motifs (Figure 7b), which contribute to the A–T richness of *A. spinosa* cp genome. This phenomenon was previously observed in cp genomes of plants species [13,25]. The SSRs were mostly detected in the non-coding regions, containing about 61.36% of total SSRs, but we also found 27.27% of SSR distributed in coding regions, such as *rpoC2* ycF2, *ndhF/G*, and *matK* and 11.36% of SSRs located in tRNA sequences. Our results are comparable to those of several previous studies showing that SSRs in cp genomes are highly rich in polythymine (polyT) or polyadenine (polyA) repeats and infrequently contain tandem cytosine (C) and guanine (G) repeats [13,28,39]. The set of SSRs identified in *A. spinosa* cp genome can be evaluated for polymorphism at the intra-specific level and used as makers for evaluating the genetic diversity among and within populations of *A. spinosa*. These markers could be also used in order to assist the selection and characterization of elite genotypes suitable for the reconstruction and extension of this endangered species.

### 2.6. Phylogenetic Inference of A. spinosa

**Figure 8:**
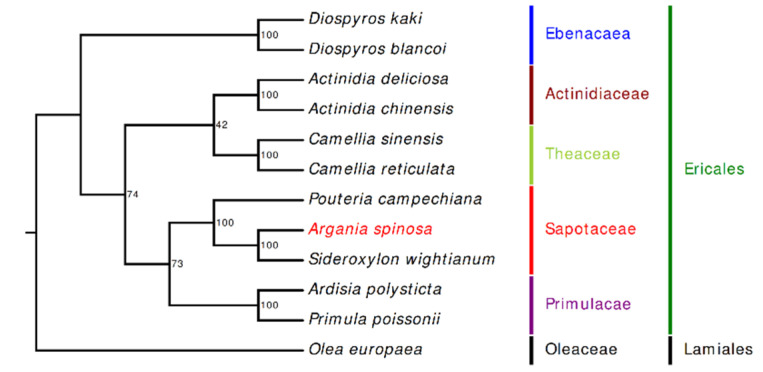
Phylogenetic tree reconstruction of 12 taxa using the maximum likelihood (ML) method based on 69 shared protein sequences. The number above each node indicates the bootstrap support values. *Olea europaea* was used as an outgroup.

In this study, 69 protein-coding genes shared by 11 members of Ericales and 1 species of Lamiales were utilized to depict the phylogenetic relationships of *A. spinosa*. Phylogenetic analyses were performed using the maximum likelihood (ML) method. The topology of the phylogenetic tree (Figure 8) separated the Sapotaceae family harboring the three species *A. spinosa, S. wightianum*, and *P. campechiana* from the neighboring families Theaceae, Primulaceae, and Acitinidiaceae. The obtained phylogenetic tree is not concordant with previous studies based on combined loci (chloroplast, mitochondrial, and nuclear), which placed the Sapotaceae close to the Ebenaceae and not to the Primulaceae [40,41]. Inconsistent topology between nuclear and plastome phylogenies has been reported for the Asterids clade and could be explained by considering several evolutionary processes such as hybridization, horizontal gene transfer, and gene duplication and loss [42]. Interestingly, the three Sapotaceae species were clustered in two clades: one clade grouping the genus *Sideroxylon* (*S. wightianum)* with *Argania* (*A. spinosa*), and the second harboring *Pouteria* (*P. campechiana*), with a strong bootstraps value (100%). In addition to the monophyletic character of the Sapotaceae members included in this study, the results highlight the close relationship of *A. spinosa* and *S. wightianum*, in accordance with the topologies inferred in precedent phylogenetic studies [7,8]. In a cladistic study of the largely tropical family Sapotaceae based on both morphological and molecular data (cp gene *ndhF*), the generated trees showed that *A. spinosa* and *Sideroxylon mascatense* attach as sisters to each other and belong to the genus *Sideroxylon* [9]. Moreover, phylogenetics inference based on 58 accessions of *trnH–psbA* and ITS sequences from *Sideroxylon* was congruent with this study [10]. Despite the indisputable fact, reported in previous studies, that *A. spinosa* is amended to *Sideroxylon*, autapomorphies characters distinguishing *Argania* from *Sideroxylon* [7,8,11] require a comprehensive exploration to confirm this relationship by including several cp genome sequences of *Sideroxylon* members close to *A. spinosa*, such as *S. mascatense*, *Sideroxylon canariense*, *Sideroxylon oxyacanthum*, and *Sideroxylon discolor*.

## 3. Materials and Methods

### 3.1. Plant Material, DNA Extraction, and Sequencing 

A single argan tree named Amghar was selected to be sequenced based on its biological and ecological proprieties. The shrub was 9 years old, with one main trunk measuring 3 m in height, and was obtained from the valley of the plain of Souss (9°32′ 00″ N, 30°24′ 00″ W; Altitude: 126 m). Genomic DNA was extracted from lyophilized leaf tissues using the Plant DNeasy mini kit according to the manufacturer’s recommendations (Qiagen, Germantown, MD, USA). Two paired-end libraries with an average insert size of 600 bp were constructed using the Nextera DNA Library Prep Kit for Illumina (New England Biolabs, New Brunswick, MA, USA) and then sequenced on the Illumina HiSeqXTen (San Diego, CA, USA) platform using 150-bp reads.

### 3.2. Chloroplast Genome Assembly 

To extract chloroplast-like reads, quality-filtered Illumina paired-end reads were mapped against the closest firstly available chloroplast genome of *P. campechiana* (taxid: 233737), using CLC Genomics (v11.0, CLCbio, Arhus, Denmark), with 0.9 and 0.95 in length and similarity, respectively. The extracted reads were de novo assembled using CLC Genomics (word size 24, bubble size 50). The generated contigs were blasted against the reference genome of *P. campechiana* (Costs: Match 2, Mismatch 3, Existence 5, Extension 2, Expectation value = 1.0 × 10^−15^, Word size = 11) to assess the assembly and to retain only the contigs aligned to the reference. The retained contigs were than assembled using the Genome Finishing Module (GFM) from CLC genomics that uses the paired-end distance information and the reference genome to order the contigs and to fill the gaps. A quadripartite structure, including IR, SCC, and LSC regions, was detected by performing a dot plot of the *A. spinosa* chloroplast sequence using the Gepard (v1.30) software (word size = 10) [43]. The validation of the assembly was performed by PCR amplification and Sanger sequencing using four couples of primers designed on the boundaries of the IR and SC regions (Table S1).

### 3.3. Genome Annotation and Comparisons

*A. spinosa* chloroplast genome was annotated through the DOGMA server [44]. The GenBank file produced was loaded into CLC genomics, and the gene list was processed manually gene by gene for the presence of start/stop codons and for internal stops in comparison to the closer cp genome of *P. campechiana*. Circular maps of the cp genome were generated using OGDraw v1.2 (https://chlorobox.mpimp-golm.mpg.de/OGDraw.html) [45]. The codon usage percentage of protein-coding sequences was estimated using the MEGA7 software [46]. Comparative genomics of *A. spinosa*, *S. wightianum*, and *P. campechiana* cp genomes was conducted using the mVISTA program in the Shuffle-LAGAN mode [47]. To calculate nucleotide diversity (Pi) between *A. spinosa, S. wightianum*, and *P. campechiana* chloroplast genomes, sliding window analysis was performed using the DnaSP version 6 software [48] with window length of 600 bp and step size of 200 bp. To assess the selective pressure on the shared protein-coding genes across the three species, the rates of synonymous (Ks) and nonsynonymous (Ka) substitutions and the Ka/Ks ratio were calculated using a Ka/Ks calculator [49].

### 3.4. Long Repetitive Sequences and Simple Sequence Repeat Analysis

Long repetitive repeat sequences, including forward, reverse, palindromic, and complement repeats, with repeat size ≥30 bp and sequence identity ≥90%, were identified using the REPuter software [50]. SSRs within the *A. spinosa* chloroplast genome were searched using the MISA software [51]. The criteria of SSR research were set to 10 repeat units as a minimum for mononucleotide repeats 5 repeat units for dinucleotides repeats, and 4 repeat units for tri- and tetranucleotides. For pentanucleotides and hexanucleotides, 3 repeats were used as the minimum.

### 3.5. Phylogenetic Analyses 

To ascertain the phylogenetic position of *A. spinosa* within the Sapotaceae family, 11 chloroplast genomes were downloaded from NCBI: *Actinidia chinensis* (NC_026690), *Actinidia deliciosa* (NC_026691), *Ardisia polysticta* (NC_021121), *Camellia reticulata* (NC_024663), *Camellia sinensis* (NC_020019), *Diospyros blancoi* (KX426216); *Diospyros kaki* (NC_030789), *Pouteria campechiana* (KX426215), *Primula poissonii* (NC_024543), *Sideroxylon wightianum* (NC_041130), and *Olea europaea* (NC_013707). Phylogenetic analysis was based on 69 protein-coding genes shared among the 12 taxa including *A. spinosa*. Individual protein sequence alignments were performed using MAFFT with default parameters [52] and then were concatenated using SeqKit with defaults parameters [53]. The whole alignment was trimmed using TrimAl [54], and the phylogeny was inferred using Randomized Axelerated Maximum Likelihood (RAxML) [55] with 1000 bootstrap replications. *O. europaea* taxon was used as an outgroup in this analysis. 

## 4. Conclusions

Comparative analyses of complete cp genomes contribute to the understanding of chloroplast structure and evolution, the identification of species, and the determination of phylogenetic relationships. In this study, we applied Illumina sequencing to determine, for the first time, the complete cp genome of the endemic species *A. spinosa*. The genome structure and genes order and content were found to be very conserved with respect to those of the close species *S. wightianum* and *P. campechiana*. Furthermore, the phylogenomic analyses based on whole cp genomes and 77 shared genes generated trees with the same topologies as previously reported, consolidating the taxonomical position of *A. spinosa* species within the Sapotaceae. To clarify the view of amending the genus *Argania* to *Sideroxylon*, it is appropriate to include more cp genomes of *Sideroxylon* members in future studies. The 44 long repeats and 88 SSRs identified here are a useful genetic resource that could be applied for population genetic studies and may also be useful for future breeding and cultivars identification. Finally, these genomic resources will certainly help in the management and conservation of this endangered species.

## Figures and Tables

**Table 1:** Features of *A. spinosa* chloroplast genome. NADH (Nicotinamide Adenine Dinucleotide Hydrogen), ORF (Open Reading Frame).

Category	Group of Genes	Name of Genes
Self-replication	Ribosomal RNAs	*rrn16*, *rrn* 23, *rrn4*.5, *rrn5*
	Transfer RNAs	*trnA-UGC*, *trnC-GCA*, *trnD-GUC*, *trnE-UUC*, *trnF-GAA*, *trnfM-CAU*, *trnG-UCC*, *trnH-GUG*, *trnI-CAU*, *trnI-GAU*, *trnK-UUU, trnL-CAA*, *trnL-UAA*, *trnL-UAG*, *trnM-CAU*, *trnN-GUU*, *trnS-GCU*, *trnP-UGG*, *trnQ-UUG*, *trnR-ACG*, *trnR-UCU trnW-CCA*, *trnY-GUA*, *trnS-GGA*, *trnS-UGA*, *trnT-GGU*, *trnT-UGU*, *trnV-GAC*, *trnV-UAC*, *trnG-GCC*
	Small subunit of ribosome	*rps2*, *rps3*, *rps4*, *rps7*, *rps8*, *rps11*, *rps12*, *rps14*, *rps15*, *rps16*, *rps18*, *rps19*
	Large subunit of ribosome	*rpl14*, *rpl16*, *rpl2*, *rpl20*, *rpl22*, *rpl23*, *rpl32*, *rpl33*, *rpl36*
	Translational initiation factor	*infA*
	DNA-dependent RNA polymerase	*rpoA*, *rpoB*, *rpoC1*, *rpoC2*
Genes for photosynthesis	NADH dehydrogenase	*ndhA*, *ndhB*, *ndhC*
		*ndhD*, *ndhE*, *ndhF*, *ndhG*, *ndhH*, *ndhI*, *ndhJ*, *ndhK*
	PS1	*psaA*, *psaB*, *psaC*, *psaI*, *psaJ*
	PS2	*psaA*, *psaB*, *psaC*, *psaI*, *psaJ*, *psbA*, *psbB*, *psbC*, *psbD*, *psbE*, *psbF*, *psbH*, *psbI*, *psbJ*, *psbK*, *psbL*, *psbM*, *psbN, psbT, psbZ*
	Cytochromeb/f complex	*petA*, *petB*, *petD*, *petG*, *petL*, *petN*
	ATP synthase	*atpA*, *atpB*, *atpE*, *atpF*, *atpH*, *atpI*
	rubisco large	*rbcL*
Other genes	maturase	*matK*
	protease	clpP
	Envelope membrane protein	cemA
	Subunit acetyl-co carboxylase	accD
	c-type cytochrome synthesis gene	*ccsA*
Genes with unknown function	ORF ycf	*Ycf1*, *ycf2*, *ycf3*, *ycf4*

## Supplementary Materials

The following are available online at https://www.mdpi.com/2223-7747/9/10/1354/s1, Figure S1: Gel electrophoresis images of PCRs performed on four different individuals of *A. spinosa* (V1, V2, V3, and V4). A and D couple of primers targeting the IRa/LSC and IRa/SSC junctions and amplifying, respectively, 700- and 258-bp fragments. B and C primers targeting the IRb/SSC and IRb/LSC junctions, amplifying, respectively, 699- and 361-bp fragments. The M lane represents the size marker. Table S1: Primers used for assembly validation of the IR/SC junction Table S2: Codon–anticodon recognition patterns and codon usage for the *A. spinosa* chloroplast genome. Table S3: Nucleotide diversity (Pi) values between the whole chloroplast genomes of *A. spinosa*, *S. wightianum*, and *P. campechiana* species using a window length of 600 bp and a step size of 200 bp, Table S4: Ka/Ks values of 79 protein-coding genes calculated for the paired sequences of the species *A. spinosa* vs. *S. wightinaum* and *A. spinosa* vs. *P. campechiana*. Table S5. Long repeats distribution within the *A. spinosa* cp genome
